# Narrative Review of the Bacterial Profile Involved in Severe COPD Exacerbations Complicated by Pneumonia in Patients Admitted to the Intensive Care Unit

**DOI:** 10.3390/jcm15197339

**Published:** 2026-09-22

**Authors:** Daniel Alexandru Jipa, Tamara Mirela Porosnicu, Patricia Hogea, Emanuela Vastag, Ovidiu Bedreag, Emanuel Ionut Poplicean, Ana Maria Mihai, Florina Cristiana Lucaciu, Cristian Oancea

**Affiliations:** 1 Doctoral School, “Victor Babes” University of Medicine and Pharmacy Timisoara, Eftimie Murgu Square 2, 300041 Timisoara, Romania; daniel.jipa@umft.ro (D.A.J.); emanuel.poplicean@umft.ro (E.I.P.); ana-maria.mihai@umft.ro (A.M.M.); florina.lucaciu@umft.ro (F.C.L.); 2 Department of Anesthesia and Intensive Care, “Victor Babes” University of Medicine and Pharmacy Timisoara, Eftimie Murgu Square 2, 300041 Timisoara, Romania; mirela.porosnicu@umft.ro (T.M.P.); bedreag.ovidiu@umft.ro (O.B.); 3 Center of Research and Innovation in Personalized Medicine of Respiratory Disease (CRIPMRD), “Victor Babes” University of Medicine and Pharmacy Timisoara, Eftimie Murgu Square 2, 300041 Timisoara, Romania; oancea@umft.ro; 4 Department of Pulmonology University Clinic, “Victor Babes” University of Medicine and Pharmacy Timisoara, Eftimie Murgu Square 2, 300041 Timisoara, Romania; 5 Department XIII, Discipline of Infectious Diseases, “Victor Babes” University of Medicine and Pharmacy Timisoara, 2 Eftimie Murgu Square, 300041 Timisoara, Romania

**Keywords:** chronic obstructive pulmonary disease (COPD), severe acute exacerbations, intensive care unit

## Abstract

Chronic obstructive pulmonary disease (COPD) is a chronic progressive respiratory disease characterized by respiratory symptoms and airflow limitation and is one of the leading causes of morbidity and mortality worldwide. The evolution of the disease is associated with episodes of acute exacerbations (AECOPD), characterized by worsening symptoms and accelerated deterioration of lung function. Acute exacerbations of COPD have different severity, being mild, moderate or severe depending on how aggravated the patients’ symptoms are, but they also depend on pulmonary functional and biological changes. Around 70% of AECOPD cases are infectious in nature, through upper or lower respiratory tract infections. Often, exacerbations associated with lower respiratory tract infections are more severe and have a higher risk of unfavorable outcome compared to AECOPD associated with upper respiratory tract infections. Severe exacerbations require hospital management, with most of them being treated in the intensive care unit (ICU). In this narrative review, we evaluated the frequency of different bacterial pathogens involved in severe COPD exacerbations associated with lower respiratory tract infections and the need for treatment in the ICU. Identifying patients with exacerbated COPD at high risk of respiratory infections, especially with Gram-negative bacilli, is extremely important to choose the optimal treatment, thus reducing the risk of death.

## 1. Introduction

Chronic obstructive pulmonary disease (COPD) is a progressive lung condition secondary to long-term exposure to harmful particles or gases. It is characterized by persistent respiratory symptoms and airflow limitation caused by damage to the airways and alveoli [1]. Worldwide, COPD is a major cause of morbidity and mortality, affecting more than 300 million people and causing over 3 million deaths annually, making it the fourth leading cause of death globally [2].

The evolution of the disease is burdened by acute exacerbations (AECOPD). According to the GOLD strategy, acute exacerbation of COPD is defined as follows: “An exacerbation of COPD is an acute event with symptoms worsening over a few days (up to 14 days) and characterized by increased dyspnea and/or cough and sputum that may be accompanied by tachypnea and/or tachycardia. Exacerbations are often associated with increased local and systemic inflammation caused by airway infection, pollution, or other insults to the lungs” [1].

Depending on the severity, exacerbations can be mild, moderate, or severe. Usually, mild to moderate exacerbations can be managed outside the hospital setting, while severe exacerbations require hospital treatment [1,3]. Admission for the management of AECOPD can be done in a clinical ward or in the intensive care unit depending on the clinical condition of the patients and the paraclinical investigations. Patients admitted to the intensive care unit (ICU) often require non-invasive ventilation (NIV), while a proportion of them may subsequently require invasive mechanical ventilation. Both forms of ventilatory support are associated with an increased risk of specific complications [4].

In this narrative review of the specialized literature, we aimed to evaluate the distribution of pathogens involved in severe COPD exacerbations that required treatment in the intensive care unit.

## 2. Materials and Methods

For the preparation of this narrative review, we searched the PubMed database for relevant articles published within the last 10 years. The search strategy included keywords and combinations of terms related to severe acute exacerbations of chronic obstructive pulmonary disease (COPD), the management of patients with COPD in the intensive care unit (ICU), and bacterial pathogens associated with severe COPD exacerbations.

Studies including patients with relevant comorbidities, such as bronchiectasis, were excluded in order to obtain more homogeneous and comparable study populations. For this narrative review, we considered studies that included patients with severe acute exacerbations of COPD requiring admission to and treatment in the ICU, including cases complicated by pneumonia or ventilator-associated pneumonia. After reviewing the eligible studies, we extracted and synthesized data on the main bacterial pathogens isolated from these patients. The findings were subsequently summarized in two tables to provide a concise overview of the bacterial etiology reported across the included studies. Only studies published in English and for which the full text was available were included in the review. We also used ChatGPT v4.0, an artificial intelligence-based language model developed by OpenAI (San Francisco, CA, USA), exclusively to create the figures based on the data presented in the review article. The entire scientific content, as well as all interpretations and conclusions, represents the authors’ original work.

## 3. Results

### 3.1. Profile of Patients with AECOPD Requiring Admission to the ICU

Patients with acute exacerbation of COPD who end up being treated in the intensive care unit (ICU) are usually those with severe exacerbation, complicated by hypercapnic respiratory failure [3]. Patients who experience such AECOPD have a poor prognosis associated with an increased risk of mortality, and either non-invasive or mechanical ventilation is used to correct respiratory failure, depending on the need [3,5]. Approximately 25% of patients with COPD exacerbation require admission to intensive care, and of these, between 26–74% require mechanical ventilator support [6,7,8].

According to the Rome Proposal, GOLD 2026, and Celli BR. et al., severe exacerbation is defined by identifying three of the following parameters regarding dyspnea on the VAS scale (visual analog dyspnea scale): respiratory rate ≥ 24 breaths/min, heart rate ≥ 95 bpm, resting SaO2 < 92% breathing ambient air or patient’s usual oxygen prescription and/or change > 3%, CRP (C-reactive protein) ≥ 10 mg/L. Also, severe exacerbation is determined if obtained ABG (arterial blood gases) show new onset/worsening hypercapnia and acidosis (PaCO2 > 45 mmHg and pH < 7.35) [1,9]. Admission to the ICU is for patients with impending or actual respiratory failure, the presence of other end-organ dysfunction (shock, renal, liver or neurological disturbance), and/or hemodynamic instability—see Figure 1 [10].

The frequency of AECOPD increases with the severity of the disease defined by the degree of airflow obstruction [1,9]. Exacerbations are also associated with disease progression and worsening respiratory dysfunction, leading to decreased quality of life and increased risk of mortality [11,12,13,14,15]. Over time, risk factors that predict relapse of exacerbations have been identified. These include a history of exacerbations; low FEV1 at diagnosis; the need for increased inhaled bronchodilator treatment or the association of corticosteroid therapy; requirement for oxygen when stable; the association of comorbidities like congestive heart failure, chronic renal, coronary artery disease, depression, anxiety or liver failure; and the use of antibiotics in the past—see Figure 1 [16,17,18,19,20,21]. However, of these predictive factors, history of exacerbations appears to be the most reliable predictor of future exacerbations [16,17]. As the number of exacerbations in the same patient increases, the severity of the exacerbation associated with the need for hospitalization also increases.

AECOPD increase the economic burden on the healthcare system, especially when they require hospitalization in the intensive care unit. Also, patients who require hospitalization for exacerbation management have a poorer prognosis and an increased risk of death compared to patients without exacerbations or those with mild exacerbations that do not require hospitalization. For these reasons, the prevention of acute events in patients with COPD represents an essential component in the management of this pathology, thus decreasing the risk of hospitalization and admission to the intensive care unit [5,10,16].

### 3.2. Bacterial Profile of Patients with AECOPD Admitted to the Intensive Care Unit

COPD exacerbations can be triggered by various factors such as respiratory infections, exposure to pollutants, previous exacerbations, associated comorbidities, or non-compliance with treatment [1,22]. Among these, respiratory infections remain the most common causes of AECOPD, representing between 70 and 80% of them. According to specialized studies, infectious exacerbations are due to bacteria in approximately 30% of cases, while viruses and bacterial–viral coinfections are involved in approximately 25% of cases each [23,24,25].

The bacterial pathogens frequently involved in AECOPD are *Haemophilus influenzae*, *Streptococcus pneumoniae* and *Moraxella catarrhalis* [25,26,27,28]. The bacterial profile changes in patients with AECOPD admitted to the intensive care unit, with patients having an increased risk of infections with multidrug-resistant bacteria such as *Acinetobacter baumannii*, *Pseudomonas aeruginosa*, *Klebsiella* spp. and *Staphylococcus aureus*—see Figure 2 [29,30,31]. The risk factors associated with the isolation of *Pseudomonas aeruginosa* or other Gram-negative bacilli in AECOPD are severe form of the disease (FEV1 < 30%), advanced age, treatment with systemic corticosteroids, administration of antibiotics in the last 3 months, and association with bronchiectasis or previous isolation of these microorganisms. Also, the involvement of these bacteria in lower respiratory tract infections in patients with COPD is associated with an increased risk of AECOPD requiring management in the hospital, including in the intensive care unit, as well as an increased risk of death [32,33,34,35,36,37,38,39].

The pathogens listed above can cause AECOPD through upper respiratory tract or lower respiratory tract infections such as pneumonia. Community-acquired pneumonia (CAP) is one of the most common respiratory infections among patients with COPD, leading to exacerbation of the disease. CAP is also a determining criterion for hospitalization of these patients, including in intensive care units, as well as a poor prognostic factor [40,41,42,43]. In patients with COPD, the pulmonary and systemic defense mechanisms are affected, which is why they are at increased risk of pneumonia compared to patients without COPD; moreover, the use of corticosteroid therapy in those with severe disease further increases the risk of respiratory infections [34,44,45].

By researching the studies in the literature, we identified several research papers that aimed to identify the pathogens involved in causing pneumonia in patients with COPD necessitating ICU admission. Most of the research aimed to identify the frequency of these bacteria in the patient groups studied.

In a prospective, multicenter study, conducted by Rello J. et al., the presence of *Streptococcus pneumoniae* was identified in 54.1% of patients with COPD and CAP admitted to the ICU, followed by *Pseudomonas aeruginosa* and *Legionella pneumophila*, which were isolated in 13.5% and 4.1% of patients, respectively. Also, in the group studied by those authors, *P. aeruginosa* predominated in patients with pneumonia and COPD, being isolated in 14.6% of patients compared to the group of patients without COPD, where it was identified in only 0.8% of patients (*p* ˂ 0.05) [46]. In agreement with this study were the results obtained by Beomsu S. et al. regarding the incidence of *S. pneumoniae* (40.3%) and *P. aeruginosa* (12.7%) in patients with COPD and CAP. Other pathogens isolated in this study in patients with severe AECOPD were *Staphylococcus aureus* in 14.2% of patients and *Klebsiella pneumoniae* in 5.2% of patients [47].

Other studies have identified *P. aeruginosa* in a smaller percentage of COPD patients. For example, in a retrospective study that pooled data over a 10-year period, *P. aeruginosa* was isolated in 7% of patients with AECOPD treated in the intensive care unit [48]. In another study, in contrast to the previously mentioned studies, *P. aeruginosa* was identified in only 2.8% of patients with AECOPD admitted to the ICU. The bacteria most frequently involved in COPD infectious exacerbations in this study were *Haemophilus influenzae* isolated in 12.8% of patients and *S. pneumoniae* in 10.8% of patients [49]. Hutanu D. et al. identified in their study that *Acinetobacter baumanii* was the most frequently isolated in patients with COPD, in a percentage of 27.4%, followed by *Klebsiella pneumoniae* in 10.7% of patients [5].

In Table 1, we have centralized the distribution of pathogens involved in severe infectious EACOPD treated in the ICU according to studies identified in the specialized literature.

In a study that aimed to assess the local epidemiological profile of respiratory infections with *Pseudomonas* and multidrug-resistant *Pseudomonas*, Adeniyi J. Idigo et al. identified a 39% higher risk of isolating *Pseudomonas* in patients diagnosed with COPD compared to those without this diagnosis. However, patients with COPD were not at risk for infections with multidrug-resistant *Pseudomonas* [50]. It appears that patients with exacerbations requiring hospitalization in the intensive care unit have a higher incidence of Gram-negative bacteria such as *Pseudomonas* and *Enterobacteriaceae* spp. compared to outpatients with AECOPD [33]. Pengwen Ouyang et al. evaluated in a retrospective, single-center study patients with infectious AECOPD secondary to pneumonia with carbapenem-resistant *Klebsiella pneumoniae*. They identified a significantly increased mortality rate among these patients compared with patients with pneumonic AECOPD of other bacterial etiology (57.69% vs. 7.69%) [51].

**Table 1:** Distribution of pathogens agents’ occurrence (%) in AECOPD with lower respiratory tract infection.

Distribution of Pathogens Agents’ Occurrence (%)	J. Rello et al., 2006 [46]	Beomsu Shin et al., 2018 [47]	Georges Abi Abdallah et al., 2024 [49]	Restrepo M.I. et al., 2006 [34]	Hutanu D et al., 2025 [5]	Mariano Rinaudo et al., 2015 [52]
*Streptococcus pneumoniae*	54.1	40.3	10.8	6.5	6	4
*Pseudomonas aeruginosa*	13.5	12.7	2.8	5.6	6	23
*Haemophilus influenzae*	11.4	0.7	12.8	3.7	-	-
*Legionella pneumophila*	4.1	0.7	0.3	-	-	-
*Acinetobacter* spp.	-	0.7	-	-	27.4	-
*Enterobacteriaceae*	3.1	-	6.6	1.9	-	8
*Klebsiella pneumoniae*	1	5.2	-	-	10.7	3
*Staphylococcus aureus*	3.1	14.2	2.1	3.2	1.2	14
*Mycobacterium tuberculosis*	2.1	-	-	-	4.8	-

### 3.3. Bacterial Profile of Patients with AECOPD and Ventilator-Associated Pneumonia

Between 26 and 76% of the patients admitted to the intensive care unit for the management of severe AECOPD require invasive mechanical ventilation (IMV) [6,7]. Some cases are complicated by ventilator-associated pneumonia (VAP), which is one of the most common complications among patients treated in intensive care and that is associated with an increased risk of morbidity and mortality, as well as a burden of treatment costs [53,54]. VAP is defined as pneumonia that occurs at least 48 h after initiation of mechanical ventilation [55].

COPD is also a known risk factor for the occurrence of VAP according to several studies conducted over time in different medical centers [52,56,57,58,59]. However, there are also studies that have shown a low incidence of VAP among patients with severe COPD exacerbation [60,61]; however, most research has shown that VAP increases morbidity and mortality in COPD patients treated in intensive care.

A recent study by Caiden Taowei Lu et al. analyzed the pathogens involved in causing VAP in patients with and without COPD over a 5-year period. The most common bacteria involved in the occurrence of VAP identified in this study were *Pseudomonas aeruginosa*, *Acinetobacter* spp., and *Klebsiella pneumoniae*, without identifying significant differences between patients with and without COPD [62]. In Gursel’s study, the pathogens responsible for VAP with the highest frequency were *Pseudomonas aeruginosa*, *Acinetobacter baumanni* and methicillin-resistant *Staphylococcus aureus* [7]. He also observed that the association of bronchiectasis with COPD increases the frequency of *Pseudomonas aeruginosa* isolation [63].

In the study conducted by Makris D et al., the bacteria most frequently involved in the occurrence of VAP in patients with AECOPD were *P. aeruginosa*, *S. aureus*, *Enterobacter* species and *A. baumannii*. Moreover, in the group of patients included in the study, mortality in the intensive care unit was significantly higher among patients with COPD compared to those without COPD (60% vs. 43.3%) [64].

Results consistent with those previously specified were also observed by Koulenti D et al. The prevalence of *P. aeruginosa* VAP was higher in patients with COPD compared to those without COPD (29.1% vs. 18.7%). Other pathogens identified in this study with increased frequency in patients with VAP were *Enterobacteriaceae*, *A. baumannii* and *Staphylococcus aureus*. The prevalence of *Enterobacteriaceae* was also higher in patients with VAP and COPD compared to those without COPD (35.4% vs. 25.9%), while for the other bacteria, the differences were not significant between the patient groups [60].

Similar results were also reported by Nseir S et al., with *P. aeruginosa* (31%), *A. baumannii* (19%) and *S. aureus* (14%) being the most common germs isolated in patients with VAP and COPD. Also, the prevalence of multidrug-resistant bacteria in these patients was found to be 41% [65].

In Table 2, we have summarized the frequency of various bacterial agents isolated in patients with COPD and VAP. Regarding the etiology of VAP in patients with COPD, Gram-negative bacilli were most frequently involved, and of these, *Pseudomonas aeruginosa* is the most often isolated.

## 4. Conclusions

This manuscript reviews the current literature regarding the clinico-bacteriological profile of patients with COPD exacerbations requiring intensive care unit management. The most common bacteria involved in severe AECOPD through lower respiratory tract infections are *Streptococcus pneumoniae* and *Pseudomonas aeruginosa*. However, we also identified studies in which *Haemophilus influenzae* or *Acinetobacter* spp. had a higher frequency in AECOPD.

Regarding the etiology of VAP, Gram-negative bacilli have been identified in most studies as determined agents, with *Pseudomonas aeruginosa* being the most frequently implicated in VAP in patients with COPD. Also, most studies have found an increase in morbidity and mortality in the intensive care unit in COPD patients who developed VAP. Further studies are needed to more comprehensively evaluate patients with severe COPD exacerbations requiring intensive care unit treatment, including studies that stratify patients according to COPD severity relative to ventilatory dysfunction. The goal of these studies could be to identify groups of patients susceptible to developing VAP so that measures to prevent VAP can be implemented.

The discrepancies among the studies reported in the literature may be explained by differences in study cohorts, demographic characteristics, severity of underlying pulmonary disease, associated comorbidities, and antibiotic treatments administered during previous exacerbations. Furthermore, patients with a history of multiple hospitalizations are at increased risk of colonization with hospital-acquired bacterial pathogens.

In clinical practice, distinguishing between contamination of biological specimens, airway colonization, and acute respiratory tract infection associated with exacerbations of pulmonary diseases, including COPD, remains an important challenge. To minimize the risk of specimen contamination, biological samples should be collected by experienced healthcare personnel. When differentiating chronic colonization from acute infection, the patient’s medical history and previous microbiological results should also be carefully reviewed. Chronic airway colonization with a specific bacterial pathogen is generally considered when the same microorganism has been isolated on three or more occasions from sputum or bronchial aspirate cultures within a six-month period. Conversely, a non-colonized status refers to the absence of persistent or recurrent isolation of a specific bacterial pathogen over time [66,67].

To establish the presence of an acute respiratory tract infection associated with a COPD exacerbation, most studies included in this review applied predefined criteria for the interpretation of microbiological samples obtained from sputum, bronchial aspirates, or blood. More specifically, the investigators considered a microorganism to be involved in the acute exacerbation when one or more of the following criteria were met:

- Sputum specimens were considered representative according to predefined quality criteria: >25 leukocytes and <10 epithelial cells per high-power field.
- Blood cultures were positive for bacterial or fungal pathogens in the absence of an extrapulmonary source of infection.
- Endotracheal aspirate cultures showed moderate or heavy growth of bacterial pathogens.
- *Mycoplasma pneumoniae* infection was diagnosed based on a fourfold increase in antibody titers between paired serum samples and/or an antibody titer of 1:160 in at least one serum sample.
- Urinary antigens for *Streptococcus pneumoniae* and *Legionella pneumophila* serogroup 1 were detected using immunochromatographic assays [5,34,46,47,49,52].

The differences observed among the analyzed studies regarding the isolation of specific bacterial pathogens may also be related to the methods used for collecting microbiological specimens, such as sputum samples versus bronchial aspirates, as well as to the diagnostic techniques employed for pathogen identification, including conventional cultures versus multiplex polymerase chain reaction (PCR) assays.

Moreover, the prevalence of bacterial pathogens involved in infectious exacerbations of COPD may vary according to geographical region and the season during which the studies were conducted. Therefore, further studies assessing temporal and geographical variations are needed to provide additional data and enhance the relevance and generalizability of the findings reported to date.

Studies aimed at mapping the pathogens most frequently involved in severe COPD exacerbations play an important role in guiding the selection of empirical antibiotic therapy while awaiting the results of sputum or bronchial aspirate cultures. Antibiotic selection in these patients should be guided by clinical presentation, disease severity, associated comorbidities, previous microbiological findings and antimicrobial susceptibility testing, the frequency and type of antibiotic exposure during previous exacerbations, and local epidemiological patterns.

The possibility of infection with Gram-negative bacteria or pathogens resistant to certain classes of antibiotics, particularly *Pseudomonas aeruginosa*, should be considered in patients with frequent exacerbations, very severe airflow obstruction, and/or a need for mechanical ventilation. Therefore, in patients with this clinical phenotype and a significant risk of *P. aeruginosa* infection, empirical antibiotic therapy with antipseudomonal activity may be warranted while awaiting microbiological results. Once culture and antimicrobial susceptibility testing results become available, antibiotic therapy should be reassessed and adjusted accordingly, with de-escalation whenever appropriate.

Further studies focusing on patients with severe COPD exacerbations requiring hospital admission, particularly those requiring management in the intensive care unit (ICU), are needed. A better understanding of the microbiological profile of severe exacerbations may facilitate earlier optimization of antimicrobial therapy and ultimately contribute to reducing mortality in this high-risk patient population.

## Figures and Tables

**Figure 1:**
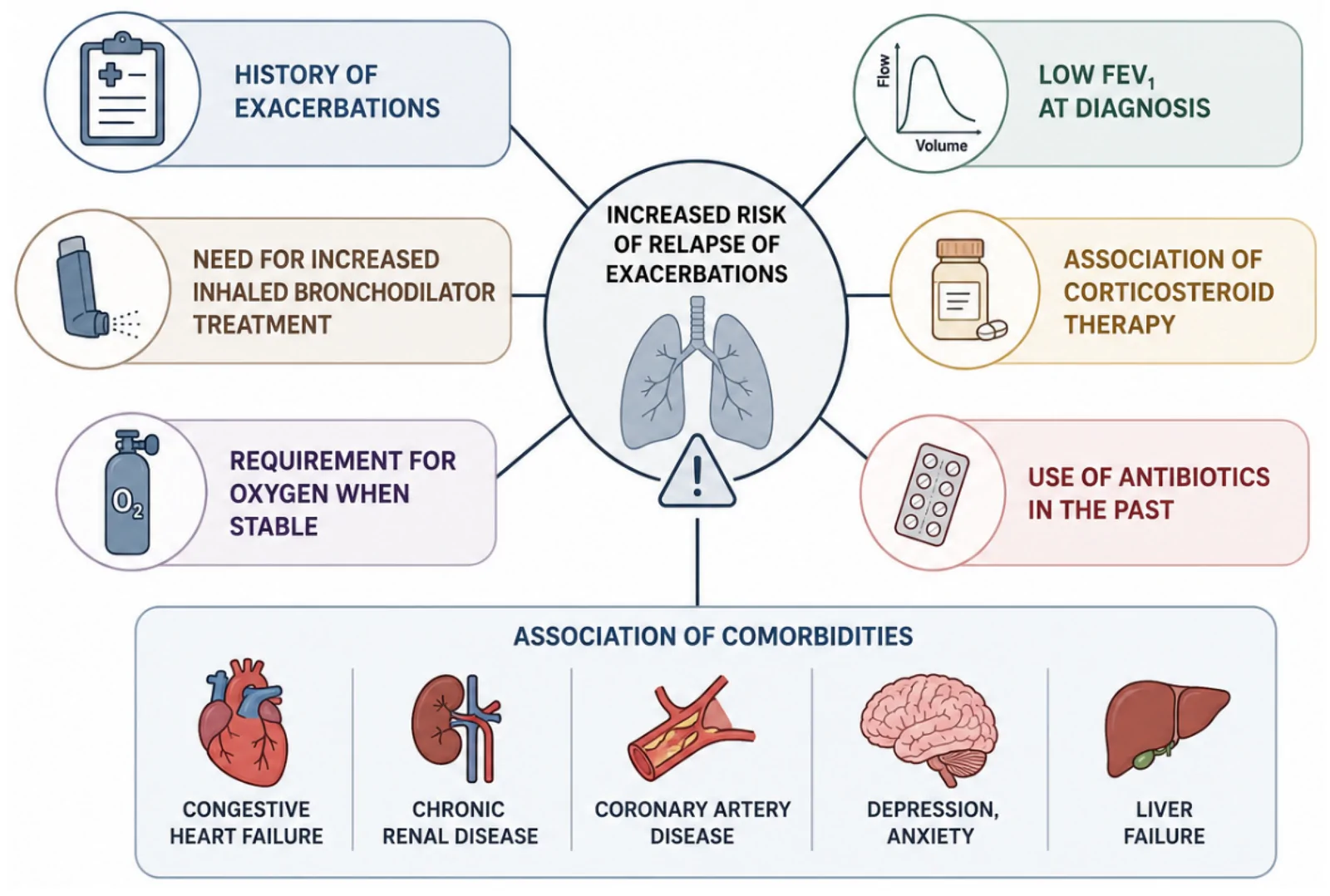
Risk factors that predict relapse of AECOPD.

**Figure 2:**
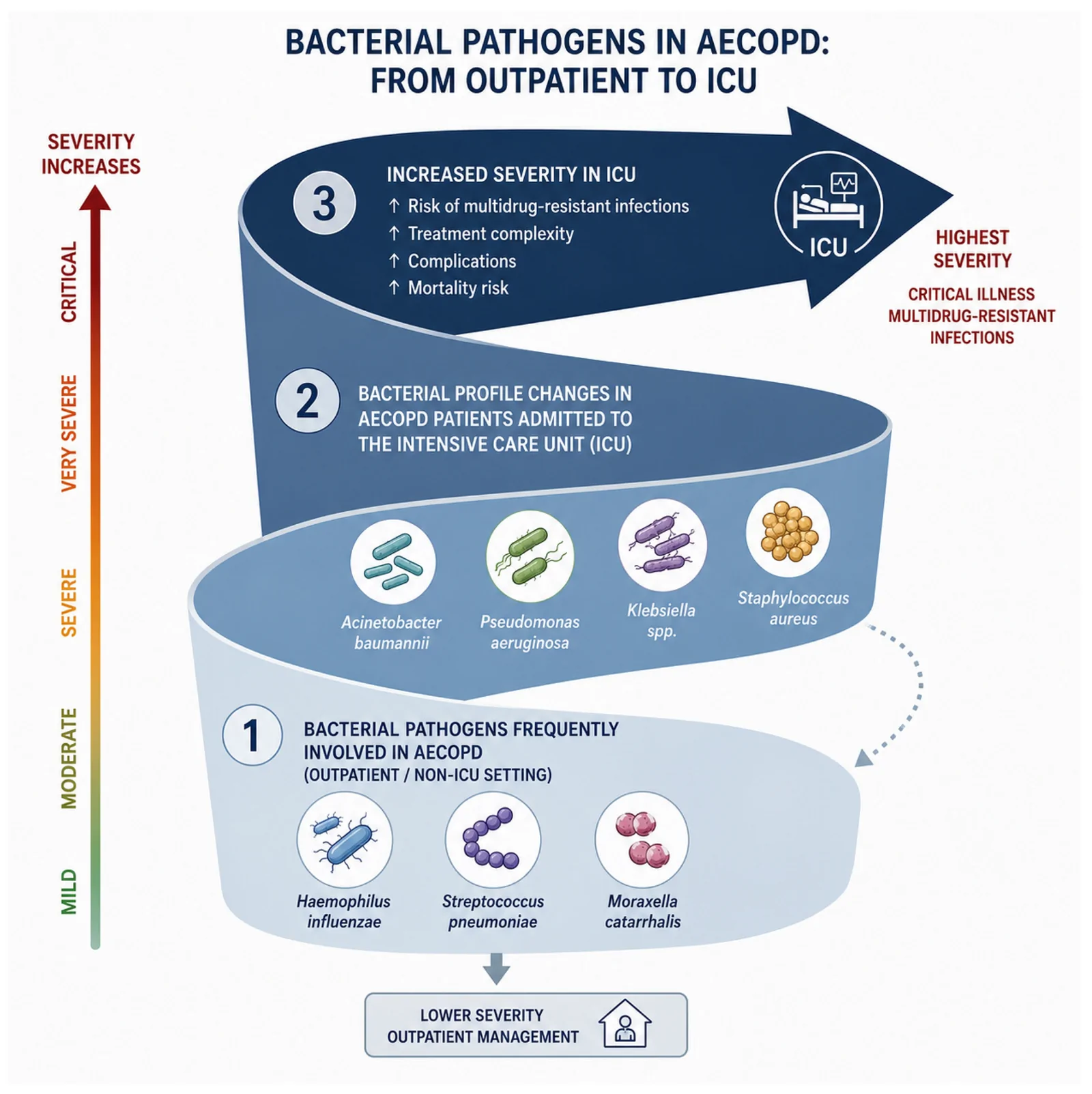
The bacterial pathogens involved in AECOPD.

**Table 2:** Distribution of pathogens agents’ occurrence (%) in patients with COPD and VAP.

Distribution of Pathogens Agents’ Occurrence (%)	Taowei C et al., 2025 [62]	Makris D et al., 2011 [64]	Koulenti D et al., 2015 [60]	Nseir S et al., 2005 [65]
*Pseudomonas aeruginosa*	19.4	33	29.1	31
*Acinetobacter* spp.	23.3	15	15.2	19
*Enterobacteriaceae*	-	12	35.4	4
*Klebsiella pneumoniae*	9.1	7	-	2
*Staphylococcus aureus*	6.5	15	19.9	13
*Stenotrophomonas maltophilia*	12.9	4	2.5	3
*Escherichia coli*	2.6	9	-	5
*Streptococcus pneumoniae*	-	-	2.5	3

## Data Availability

Since this is not a study in which the authors directly monitored these patients—and there is no available database—all the data used were analyzed based on other reference studies.

## Abbreviations

The following abbreviations are used in this manuscript:

ABG	Arterial blood gases
AECOPD	Acute exacerbations of chronic obstructive pulmonary disease
COPD	Chronic obstructive pulmonary disease
CAP	Community-acquired pneumonia
CRP	C-reactive protein
GOLD	Global initiative for chronic obstructive lung disease
ICU	Intensive care unit
IMV	Invasive mechanical ventilation
SaO2	Oxygen saturation
VAP	Ventilator-associated pneumonia
VAS	Visual analog dyspnea scale
